# Clinical features of macrophage activation syndrome in adult dermatomyositis: A single‐center retrospective case‐control study

**DOI:** 10.1002/iid3.1141

**Published:** 2024-01-10

**Authors:** Dingxian Zhu, Shuni Ying, Changyi Yang, Sheng Li, Shunli Tang, Chuanyin Sun, Hong Fang, Jianjun Qiao

**Affiliations:** ^1^ Department of Dermatology, The First Affiliated Hospital Zhejiang University School of Medicine Hangzhou China; ^2^ Department of Rheumatology, The First Affiliated Hospital Zhejiang University School of Medicine Hangzhou China

**Keywords:** complication, dermatomyositis, macrophage activation syndrome, rapidly progressive interstitial lung disease

## Abstract

**Background:**

Little is known about the features of macrophage activation syndrome (MAS) in dermatomyositis, especially the association between rapidly progressive interstitial lung disease (RP‐ILD) and MAS.

**Objective:**

To determine the characteristics of MAS in patients with dermatomyositis and their association with RP‐ILD.

**Methods:**

This was a retrospective cohort study of 201 dermatomyositis patients at the First Affiliated Hospital of Zhejiang University over a 10‐year period.

**Results:**

A total of 22 (10.9%) patients were diagnosed with MAS. The rate of RP‐ILD was significantly higher in patients with MAS than in those without MAS (81.8% vs. 17.4%, respectively, *p* < .001). Multivariate analysis indicated that RP‐ILD (*p* = .019), ferritin level > 1685 ng/mL (*p* = .007) and hemoglobin < 100 g/L (*p* = .001) were independent risk factors for MAS. Furthermore, RP‐ILD patients with MAS presented more cardiac injury (50.0% vs. 13.3%, respectively, *p* < .009), central nervous system dysfunction (42.8% vs. 3.4%, respectively, *p* < .001) and hemorrhage (38.9% vs. 3.3%, respectively, *p* = .003) than RP‐ILD patients without MAS. The 90‐day cumulative survival rate for patients with MAS was significantly lower than for those without MAS (18.2% vs. 82.1%, respectively, *p* < .001).

**Conclusion:**

MAS was a common and fatal complication of dermatomyositis in our cohort. MAS is closely related to RP‐ILD in patients with dermatomyositis. When RP‐ILD is present in dermatomyositis patients with abnormal laboratory findings, such as cytopenia and hyperferritinemia, the presence of MAS should be considered.

## Highlights

- This study adds evidence that macrophage activation syndrome (MAS) is a common and fatal complication of adult dermatomyositis in our cohort. MAS is closely related to RP‐ILD in patients with dermatomyositis.
- Clinicians should be aware that when RP‐ILD is present in dermatomyositis patients with abnormal laboratory findings, such as cytopenia and hyperferritinemia, the presence of MAS should be considered.

## Graphical abstract

(1) This study adds evidence that macrophage activation syndrome (MAS) is a common and fatal complication of adult dermatomyositis in our cohort. MAS is closely related to RP‐ILD in patients with dermatomyositis. (2) Clinicians should be aware that when RP‐ILD is present in dermatomyositis patients with abnormal laboratory findings, such as cytopenia and hyperferritinemia, the presence of MAS should be considered.

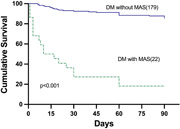

## 1 INTRODUCTION

Dermatomyositis is an autoimmune disease characterized by skin lesions and skeletal myopathy, with an estimated prevalence of 9.63 per 1 million persons in the general population.^1^ One of the most intractable and life‐threatening complications of dermatomyositis is the occurrence of rapidly progressive interstitial lung disease (RP‐ILD), which is extremely difficult to treat and related to a high mortality rate, with a 6‐month survival rate of only 41%.^2^ The pathogenesis of RP‐ILD patients is still not clear; histopathological examinations of the lung tissues in RP‐ILD patients have revealed the presence of infiltrating alveolar macrophages.^3^ Furthermore, the involvement of cytokines associated with macrophage activation, such as interleukin‐6 (IL‐6), interleukin‐18 (IL‐18), and interferon‐gamma (IFN‐γ), may contribute to its pathogenetic mechanisms.^4^ Moreover, there is documented evidence establishing a significant association between neopterin, a macrophage activation marker, and RP‐ILD, which suggesting its potential role as a biomarker in the disease evaluation of dermatomyositis.^5^

Haemophagocytic syndrome, also called hemophagocytic lympho­histiocytosis (HLH), is a rare immune‐mediated life‐threatening disease, caused by a dysregulated hyperinflammatory response, associated with aberrant activation of lymphocytes and macrophages, leading to a severe hyperinflammatory condition.^6^ The secondary form of HLH during rheumatic disorders is called macrophage activation syndrome (MAS), which is specifically associated with autoimmune diseases such as systemic‐onset juvenile idiopathic arthritis and adult‐onset Still's disease.^7^, ^8^ This syndrome is characterized by fever, hepatosplenomegaly, hyperferritinemia, cytopenia, coagulopathy, hypertriglyceridemia, and hemophagocytic cells in the bone marrow.^8^ Additionally, elevated serum ferritin, a well‐established biomarker for dermatomyositis‐associated RP‐ILD, constitutes a notable laboratory abnormality in the diagnosis and assessment of MAS.^9^, ^10^

We have seen some patients exhibiting hyperferritinemia, which is accompanied by cytopenia, and hypofibrinogenemia, which represents atypical clinical manifestations in dermatomyositis. These patients tend to be more severe and often present with RP‐ILD. Taken together, we considered MAS may be a complication of dermatomyositis. However, little is known about the features of MAS in dermatomyositis, especially the association between RP‐ILD and MAS.

In this study, we performed a retrospective study in a tertiary hospital in China over a 10‐year period and analyzed the characteristics of MAS in patients with dermatomyositis. The relationship between MAS and RP‐ILD was also revealed.

## 2 METHODS

### 2.1 Patients and study design

We retrospectively analyzed the clinical and laboratory data of 279 patients with active dermatomyositis who were initially admitted to the First Affiliated Hospital of Zhejiang University from January 2010 to August 2020. The diagnosis of dermatomyositis was made according to the Bohan and Peter criteria.^11^ The exclusion criteria were lack of data; overlap syndromes, including systemic lupus erythematosus, rheumatoid arthritis, scleroderma, and Sjögren syndrome; and age <18 years. RP‐ILD was defined as a previous or concurrent diagnosis of ILD and acute and progressive worsening of dyspnea secondary to ILD requiring hospitalization, supplementary oxygen, or respiratory failure requiring intubation within 3 months of the diagnosis of ILD.^12^ This study was performed with the approval of the Human Ethics Board of the First Affiliated Hospital, Zhejiang University School of Medicine (approval no: 2020‐ITT‐775).

To increase the accuracy and sensitivity of MAS diagnosis, we used two diagnostic criteria, the HLH‐2009 diagnostic criteria^13^ and the HScore.^14^ Patients who met both the HLH‐2009 diagnostic criteria and had an HScore higher than 169 were diagnosed with MAS. All diagnoses were confirmed by at least two specialists (CS and ST).

Patients' clinical, histopathological, lung imaging, and laboratory data were collected. Laboratory findings on admission included complete blood count, serum creatinine, liver function tests, serum ferritin, lactate dehydrogenase, triglyceride, and fibrinogen levels. Some laboratory tests of MAS may not have been done on the same day, so we selected the laboratory findings within 3 days of the date of laboratory data selection for the diagnosis of MAS. The time from dermatomyositis symptom onset to MAS diagnosis was determined. All patients were followed up for at least 1 year to assess survival. The MYOACT scores were used to assess the disease activity of dermatomyositis.^15^

### 2.2 Statistical analysis

We compared the features of dermatomyositis patients with MAS to those without MAS using the Student's *t* test, the Mann–Whitney *U* test, and the chi‐square test. Fisher's exact test was used to identify and evaluate the frequency of complications between dermatomyositis patients with RP‐ILD with and without MAS. The cumulative survival rate was calculated using the Kaplan–Meier test. The log‐rank test was also used to compare survival. In all analyses, *p* < .05 was taken to indicate statistical significance.

## 3 RESULTS

### 3.1 Demographic, clinical, and laboratory features of MAS in patients with dermatomyositis

A total of 279 cases of patients with active dermatomyositis were analyzed, and 78 patients were excluded due to a lack of information or overlap syndromes. Therefore, a total of 201 dermatomyositis patients were finally included in the study. Of these 201 adult dermatomyositis patients, 22 (10.9%) both met the HLH‐2009 classification criteria, and an HScore higher than 169 was diagnosed with MAS (Table 1). The demographic, clinical, and laboratory features of the 22 patients with MAS are shown in Table 2.

**Table 1:** The proposed HLH criteria, 2009 and Hscore.

Symptoms	Cases																					
	1	2	3	4	5	6	7	8	9	10	11	12	13	14	15	16	17	18	19	20	21	22
1. Molecular diagnosis of HLH or XLP	No	No	No	No	No	No	No	No	No	No	No	No	No	No	No	No	No	No	No	No	No	No
2. Or at least three of four																						
a. Fever	Yes	Yes	Yes	Yes	Yes	Yes	Yes	Yes	Yes	Yes	Yes	Yes	Yes	Yes	Yes	Yes	Yes	Yes	Yes	Yes	Yes	Yes
b. Splenomegaly	Yes	Yes	Yes	No	Yes	Yes	No	No	Yes	No	No	Yes	Yes	Yes	Yes	No	No	No	Yes	No	Yes	Yes
c. Cytopenias	Yes	Yes	Yes	Yes	No	Yes	Yes	Yes	Yes	Yes	Yes	Yes	No	Yes	Yes	Yes	Yes	Yes	Yes	Yes	Yes	No
d. Hepatitis	Yes	Yes	No	Yes	Yes	Yes	Yes	Yes	Yes	Yes	Yes	Yes	Yes	Yes	Yes	Yes	Yes	Yes	Yes	Yes	Yes	Yes
3. In addition, at least one of four:																						
a. Hemophagocytosis	No	Yes	Yes	No	No	No	No	No	No	No	No	No	No	No	No	No	No	No	No	No	No	No
b. Ferritin	Yes	Yes	Yes	Yes	Yes	Yes	Yes	Yes	Yes	Yes	Yes	Yes	Yes	Yes	Yes	Yes	Yes	Yes	Yes	Yes	Yes	Yes
c. sIL2Ra (age‐based)	‐	‐	‐	‐	‐	‐	‐	‐	‐	‐	‐	‐	‐	‐	‐	‐	‐	‐	‐	‐	‐	‐
d. Absent or much decreased NK function	‐	‐	‐	‐	‐	‐	‐	‐	‐	‐	‐	‐	‐	‐	‐	‐	‐	‐	‐	‐	‐	‐
4. Other results supportive of HLH Diagnosis																						
a. Hypertriglyceridaemia	No	Yes	No	Yes	Yes	No	No	Yes	Yes	Yes	No	No	Yes	No	No	Yes	No	No	Yes	Yes	Yes	Yes
b. Hypofibrinogenemia	Yes	Yes	Yes	Yes	Yes	Yes	Yes	Yes	Yes	No	Yes	Yes	Yes	Yes	Yes	Yes	Yes	Yes	Yes	Yes	Yes	Yes
c. Hyponatremia																						
Diagnosed by these criteria	Yes	Yes	Yes	Yes	Yes	Yes	Yes	Yes	Yes	Yes	Yes	Yes	Yes	Yes	Yes	Yes	Yes	Yes	Yes	Yes	Yes	Yes
Hscore	172	176	266	188	232	256	245	223	228	199	185	241	252	203	197	197	180	182	237	216	243	205

Abbreviations: HLH, hemophagocytic lympho­histiocytosis; NK, natural killer.

**Table 2:** Clinical and laboratory features of 22 patients with dermatomyositis with MAS.

Features	Patients no. (%)	Mean ± SD
Female gender, *n* (%)	14 (63.6)	‐
Age at onset of MAS (years)	50.27 ± 3.25	‐
MAS as onset of DM, *n* (%)	14 (63.6)	‐
Duration of DM at MAS onset (months)	4.25	‐
Fever^a^	22 (100)	‐
Hepatomegaly^a^	3 (13.6)	‐
Splenomegaly^a^	14 (63.6)	‐
Lymphadenopathy	10/17 (58.8)	‐
Cytopenia		
Neutrophils <1.0 × 10^9^/L^a^	5/22 (22.7)	10.67 ± 2.02
Hemoglobin <90 g/L^a^	19/22 (86.4)	73.6 ± 4.41
Platelets <100 × 10^9^/L^a^	19/22 (86.4)	60.6 ± 13.28
Liver dysfunction		
ALT >40 U/L	18/22 (81.8)	136.54 ± 21.44
AST >40 U/L	20/22 (90.9)	206.9 ± 32.44
LDH >250U/L	22 (100)	1033.45 ± 190.31
Fibrinogen ≤1.5 g/L^a^	19/21 (90.5)	1.16 ± 0.09
Triglycerides >3.0 mmol/L^a^	11/19 (57.9)	4.63 ± 0.77
Complications		
Heart failure, *n* (%)	8/22 (36.4)	‐
Respiratory failure, *n* (%)	18/22 (81.8)	‐
Hemorrhage, *n* (%)	8/22 (36.4)	‐
CNS dysfunction, *n* (%)	8/22 (36.4)	‐
ICU admission, *n* (%)	15/22 (68.1)	‐
Death, *n* (%)	18/22 (81.8)	
Time to death after MAS (days)	10.2	

Abbreviations: ALT, Alanine aminotransferase; AST, aspartate aminotransferase; CNS, central nervous system; DM, dermatomyositis; ICU, intensive care unit; LDH, lactate dehydrogenase; MAS, macrophage activation syndrome.

^a^ Criteria of MAS.

Fever and hyperferritinemia in all cases, hypofibrinogenemia and hepatitis in 21 cases, cytopenias in 19 cases, splenomegaly in 13 cases, and hypertriglyceridemia in 12 cases were found (Table 1). In patients with MAS, 18 (81.8%) patients developed respiratory failure, and 8 (36.4%) patients presented heart failure, with elevated BNP levels (599–9000 pg/mL). In eight (36.4%) patients with central nervous system dysfunction, five patients developed drowsiness and apathia, two had mania and hallucination, and one had cerebral infarction. In eight (36.4%) patients with hemorrhage, six patients developed gastrointestinal bleeding, one had intracerebral hemorrhage, and one had intrahepatic hemorrhage.

**Figure 1:**
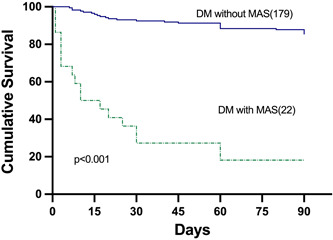
Cumulative survival rates of dermatomyositis (DM) patients with and without MAS for 90 days. The cumulative survival rate was significantly lower in patients with MAS than in those without MAS (18.2% vs. 82.1%, *p* < .001). MAS, macrophage activation syndrome.

MAS occurred in the early stage of dermatomyositis (<15 days) in 14 of the 22 patients (63.6%). The short‐term mortality rate for MAS patients was 81.8%, and the ICU admission was 68.1% (Figure 1 and Table 2). The mean time to death after MAS was 10.2 days (Table 2).

**Figure 2:**
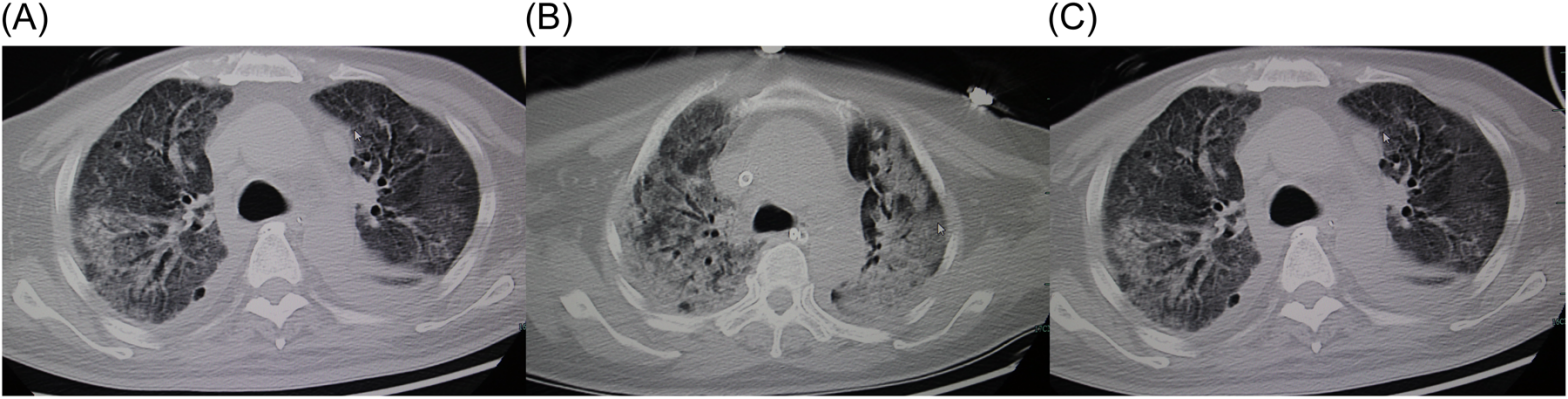
RP‐ILD of three MAS patients with dermatomyositis. MAS, macrophage activation syndrome; RP‐ILD, rapidly progressive interstitial lung disease.

### 3.2 Comparison of clinical and laboratory features between dermatomyositis patients with and without MAS

Table 3 shows the comparison between dermatomyositis patients with and without MAS. The two groups were matched for age and sex. Patients with MAS showed higher rates of fever (100% vs. 28.5%, respectively, *p* < .001) and Gottron's sign (40.9% vs. 16.8%, respectively, *p* = .018). Patients with MAS showed higher rates of infection (72.7% vs. 25.2%, respectively, *p* <  .001) and higher disease activity, which was evaluated by MYOACT (*p* < .001). Patients with MAS use more high‐dose methylprednisolone (*p* = .002) and IVIG (*p* = .001). All other clinical features were comparable between the two groups. With regard to laboratory findings, alanine aminotransferase (ALT), aspartate aminotransferase (AST), lactate dehydrogenase (LDH), and ferritin levels were higher, while the hemoglobin level, platelet count, and fibrinogen level were markedly lower in dermatomyositis patients with MAS. However, there were no significant differences in white blood cell (WBC) or neutrophil counts or triglyceride levels between the two groups. Moreover, patients with MAS were more likely to present with splenomegaly (63.6% vs. 5.2%, respectively, *p* < .001) and had a significantly higher rate of RP‐ILD than those without MAS (81.8% vs. 17.4%, respectively, *p* < .001) (Table 3 and Figure 2).

**Table 3:** Comparison of clinical manifestations and laboratory features between patients with dermatomyositis with and without MAS.

	DM with MAS (*n* = 22)	DM without MAS (*n* = 179)	*p* Value
Age, mean ± SD (years)	50.32 ± 3.25	54.83 ± 0.99	.14
Female gender, *n* (%)	14 (63.6)	108 (60.3)	.8
Skin eruption			
Heliotrope rash, *n* (%)	8 (36.3)	85 (47.5)	.371
Gottron papules, *n* (%)	12 (54.5)	68 (37.9)	.167
Gottron sign, *n* (%)	9 (40.9)	30 (16.8)	.018*
Mechanic's hands, *n* (%)	1 (4.5)	12 (6.7)	.689
Poikiloderma, *n* (%)	2 (9.0)	6 (3.3)	.214
V‐neck rash, *n* (%)	4 (18.1)	61 (34.0)	.154
Shawl sign, *n* (%)	4 (18.1)	32 (17.8)	.972
Skin ulcers, *n* (%)	1 (4.5)	10 (5.5)	.839
Physical findings			
Fatigue, *n* (%)	11 (50.0)	108 (60.3)	.364
Fever, *n* (%)^a^	22 (100)	51 (63.6)	<.001***
Arthralgia, *n* (%)	4 (18.1)	48 (28.5)	.451
Shortness of breath, *n* (%)	2 (9.0)	38 (21.2)	.259
Dysphagia, *n* (%)	7 (31.8)	37 (20)	.233
Laboratory findings			
White blood cell (10^9^ /L)	12.11 ± 2.26	8.33 ± 0.35	.113
Neutrophils cell (10^9^ /L)^a^	10.67 ± 2.02	6.46 ± 0.31	.051
Hemoglobin (g/L)^a^	77.22 ± 4.45	120.39 ± 1.32	<.001***
Platelet (10^9^/L)^a^	67.95 ± 10.38	192.78 ± 5.96	<.001***
Ferritin level, mean ± SD (ng/mL)^a^	9303.68 ± 2770.47	1140.82 ± 179.73	.008**
ALT, mean ± SD (U/L)^a^	136.54 ± 21.44	68.92 ± 6.17	.006**
AST, mean ± SD (U/L)^a^	206.86 ± 32.44	90.22 ± 9.58	<.001***
CK, mean ± SD (U/L)	789.95 ± 263.04	1117.78 ± 143.58	.282
LDH, mean ± SD (U/L)	1033.45 ± 190.31	472.26 ± 19.15	<.001***
Fibrinogen (g/L)^a^	1.16 ± 0.09	3.14 ± 0.08	<.001***
Triglycerides (mmol/L)^a^	4.62 ± 0.73	2.92 ± 0.83	.499
ESR (mm/H)	23 ± 5.52	28.42 ± 1.82	.303
CRP (mg/L)	30.11 ± 8.94	17.74 ± 2.42	.194
Complications			
ILD, *n* (%)	18 (81.8)	88 (49.2)	.037
RP‐ILD, *n* (%)	18 (81.8)	30/172 (17.4)	<.001***
Splenomegaly, *n* (%)^a^	14 (63.6)	7/133 (5.2)	<.001***
Carcinoma, *n* (%)	4 (18.2)	39 (21.8)	.697
Infection, *n* (%)	16 (72.7)	41 (25.2)	<.001***
Disease activity			
MYOACT	6.4 ± 0.44	2.39 ± 0.12	<.001***
ANA	7 (33.3)	136 (77.3)	<.001***
Anti‐SSA	1 (4.8)	33 (18.6)	.111
Anti‐SSA52	6 (28.6)	68 (38.4)	.378
Anti‐SSB	0 (0)	12 (6.8)	.218
Anti‐JO1	0 (0)	11 (37.8)	.068
Medication			
Glucocorticoids	22 (100)	167 (93.3)	.235
Low‐dose methylprednisolone	1 (4.5)	24 (13.4)	.21
Middle‐dose methylprednisolone	6 (27.3)	87 (48.6)	.048
High‐dose methylprednisolone	14 (63.6)	54 (30.2)	.002**
Disease‐modifying anti‐rheumatic	11 (50)	70 (39.1)	.326
Drugs			
Hydroxychloroquine	1 (4.5)	12 (6.7)	.689
Thalidomide	0 (0)	25 (14.0)	.061
Methotrexate	1 (4.5)	1 (0.6)	.075
Cyclosporine	0 (0)	5 (2.8)	.427
IVIG	9 (40.9)	5 (2.8)	.001**

*Note*: The data in this table were tested at the onset of MAS.

Abbreviations: ALT, alanine aminotransferase; AST, aspartate aminotransferase; CRP, C‐reactive protein; DM, dermatomyositis; ESR, erythrocyte sedimentation rate; IVIG, intravenous immunoglobulin; LDH, lactate dehydrogenase; MAS, macrophage activation syndrome; RP‐ILD, rapidly progressive interstitial lung disease.

^a^ Criteria of MAS.

* *p* < .05

** *p* < .01

*** *p* < .001.

### 3.3 Predictive factors of MAS in patients with dermatomyositis

Univariate and multivariate Cox proportional hazard regression models were used to identify risk factors for the occurrence of MAS in patients with dermatomyositis (Table 4). Univariate Cox regression analysis showed that fever, Gottron's sign, hemoglobin <100 g/L, platelet count <90 × 10^9^/L, ferritin >1685 ng/mL, ALT >200 U/L, AST >200 U/L, fibrinogen <1.5 g/L, RP‐ILD, ANA, infection, and splenomegaly had predictive significance (all *p* < .05). Multivariate Cox analysis confirmed that a ferritin level >1685 ng/mL, RP‐ILD, and a hemoglobin level <100 g/L were independent predictive factors for MAS.

**Table 4:** Univariate and multivariate analysis of predictive factors for MAS in patients with dermatomyositis.

	Univariate analysis			Multivariate analysis		
Variable	HR	95% CI	*p* Value	HR	95% CI	*p* Value
Fever	48	6.37–370.61	<.001***			
Gottron sign	3.44	1.35–8.77	.01**			
Hemoglobin <100 g/L	47.65	12.99.13–174.84	<.001***	62.24	5.45–711.20	.001**
Platelet <90 × 10^9^/L	23.85	8.29–68.651	<.001***			
Ferritin level >1685 ng/mL	29.00	8.54–98.48	<.001***	21.74	2.30–205.33	.007*
ALT >200 U/L	5.63	2.22–14.28	<.001***			
AST >200 U/L	8.69	3.35–22.54	<.001***			
LDH >1000 U/L	2.99	3.35–22.54	.055			
Fibrinogen <1.5 g/L	117.86	24.95–556.71	<.001***			
RP‐ILD	22.35	7.06–70.74	<.001***	13.12	1.52–113.26	.019*
Splenomegaly	31.50	9.25–99.97	<.001***			
ANA	0.14	0.056–0.38	<.001***			
Infection	7.93	2.911–21.62	<.001***			
MYOACT >5	11.55	4.32–30.90	<.001***			
IVIG	4.696	1.805–12.215	<.001***			

Abbreviations: ALT, alanine aminotransferase; AST, aspartate aminotransferase; IVIG, intravenous immunoglobulin; LDH, lactate dehydrogenase; MAS, macrophage activation syndrome; RP‐ILD, rapidly progressive interstitial lung disease.

* *p* < .05

** *p* < .01

*** *p* < .001.

### 3.4 Comparison of complications between RP‐ILD dermatomyositis patients with and without MAS

RP‐ILD patients with MAS had a higher frequency of cardiovascular complications (50.0% vs. 13.3%, respectively, *p* < .009) and a higher frequency of central nervous system involvement than those without MAS (42.8% vs. 3.4%, respectively, *p* < .001). There was also a significant difference in the rate of hemorrhage between the two groups (38.9% vs. 3.3%, respectively, *p* = .003). However, there were no significant differences in liver failure or kidney involvement between the two groups. The mortality rate and rate of ICU admission were significantly higher in the MAS group than in the non‐MAS group (*p* < .001, Table 5).

**Table 5:** Comparison of complications between RP‐ILD dermatomyositis patients with and without MAS.

Organ damage	RP‐ILD with MAS	RP‐ILD without MAS	*p* Value
Cardiac injury	9/18 (50%)	4/30 (13.3%)	.009*
Liver failure	8/18 (44.4%)	8/30 (26.7%)	.226
Central nervous system dysfunction	9/18 (42.8%)	1/30 (3.4%)	<.001***
Hemorrhage	8/18 (44.4%)	1/30 (3.3%)	.003**
Kidney involvement	3/18 (16.7%)	6/30 (20.0%)	.735
ICU admission	14/18 (85.7%)	6/30 (20.0%)	<.001***
Mortality	16/18 (88.9%)	13/30 (43.3%)	.008**

Abbreviations: ICU, intensive care unit; MAS, macrophage activation syndrome; RP‐ILD, rapidly progressive interstitial lung disease.

* *p* < .05

** *p* < .01

*** *p* < .001.

### 3.5 Survival

The 90‐day cumulative survival rate was 82.1% for dermatomyositis patients without MAS but only 18.2% in patients with dermatomyositis and MAS (*p* < .001) (Figure 1). The mortality rate in the RP‐ILD patients with MAS group was significantly higher than the RP‐ILD patients without MAS group(88.9% vs. 43.3%, *p* < .01, Table 5).

## 4 DISCUSSION

In the present study, we have demonstrated for the first time that 10.9% of dermatomyositis patients were complicated with MAS. Consistent with our result, MAS may occur in patients with dermatomyositis but has only been described previously in case reports^16^, ^17^, ^18^, ^19^, ^20^ and small case series.^21^, ^22^ Our results highlight the clinical and laboratory features and the high mortality rate of MAS in adult dermatomyositis patients. In addition, we have provided rigorous evidence that patients with RP‐ILD are more susceptible to MAS and the presence of MAS may cause multiple‐organ multiple organ failure.

The current classification of MAS is still imprecise. Currently used criteria include HLH‐2004,^23^ HLH‐2009‐0.52^13^ 2016 Ravelli criteria,^24^ and Hscore.^14^ Since MAS is a subtype of HLH, the HLH‐2004 guideline is currently the most widely used to diagnose MAS. However, tests such as natural killer (NK) cell activity and sCD25 levels in the HLH‐2004 diagnostic criteria were not measured regularly in our hospital. Some MAS cases will be false negative and incorrectly diagnosed as “normal” cases. Therefore, we did not use the HLH‐2004 diagnostic criteria. In our study, we used two different diagnostic criteria: the HLH‐2009 diagnostic criteria and the Hscore. The diagnosis of MAS was also confirmed when at least both criteria were met at the same time. In our cohort, the application of the HLH‐2009 diagnostic criteria poses inherent challenges. Despite the inclusion of NK cell activity and sCD25 levels in the HLH‐2009 criteria, their impact on the final diagnosis is notably diminished. The utilization of AST and ALT as markers for hepatitis within the HLH‐2009 diagnostic criteria may lead to the inclusion of diagnoses that would not otherwise meet the criteria for MAS. Although research suggests that elevated liver function in dermatomyositis with MDA5 positivity may be associated with macrophage activation, which may be linked to the pathological mechanisms of MAS.^25^ Considering that abnormal liver function in dermatomyositis can, in part, be attributed to elevated muscle enzymes, we opted for a more stringent diagnostic approach. Therefore, we augmented the HLH‐2009 criteria with the Hscore diagnostic criteria. Moreover, two experts were involved in evaluating and comparing laboratory test results before and after the onset of MAS. This process aimed to confirm the diagnosis, enhancing diagnostic precision. In our cohort, the clinical characteristics of MAS in dermatomyositis may be different from MAS in other diseases. Thus, to timely diagnosis, a new MAS diagnostic criterion developed in the context of dermatomyositis should be established.

In our cohort, the occurrence of MAS in dermatomyositis is not associated with malignant tumors or infections. Instead, it is closely related to the development of RP‐ILD. Notably, the pathogenic mechanisms underlying these two conditions share overlapping features.

First, the activation of macrophages was involved in both of them. Previous investigations have established the presence of activated macrophages in the pulmonary parenchyma of RP‐ILD patients.^3^ In dermatomyositis, autoimmune stimuli incite the activation of alveolar macrophages, prompting the secretion of a spectrum of cytokines, including IL‐6 and IFN‐γ. These mediators, in turn, stimulate neutrophil recruitment and contribute to the induction of pulmonary fibrosis.^4^, ^26^ Similarly, the pathogenesis of MAS is intricately linked to the excessive activation and proliferation of macrophages.^27^ Aberrant macrophage activation, coupled with an elevated cytokine release, involves a complex interplay of multiple cell types and cytokines, culminating in an escalating cytokine production and the onset of a cytokine storm.^28^

Second, both conditions are characterized by hyperferritinemia. Serum ferritin is an important laboratory hallmark in MAS.^10^ Mechanistically, ferritin, as a pathogenic mediator, triggers pro‐inflammatory mediators, leading to an immune response and inducing the cytokine storm in MAS.^29^ In dermatomyositis, elevated ferritin is also a biomarker for predicting RP‐ILD. These elevated ferritin levels are also closely associated with the activation of alveolar macrophages and elevated pro‐inflammatory factors including IL‐6 and IFN‐γ.^30^ Consequently, recent evidence suggests that, in addition to adult‐onset Still's disease and MAS, MDA5‐positive dermatomyositis should be included within the spectrum of hyperferritinemia syndrome.^29^ In summary, in patients with dermatomyositis concomitant with MAS, macrophage activation leads to a high‐immune response resulting in an elevated inflammatory state and a cytokine storm.

Although dermatomyositis‐associated RP‐ILD and MAS share some similarities, there are still differences. Our data indicates that compared to patients with isolated RP‐ILD, nervous system dysfunction was present in 42.8% of MAS patients, 50% of patients experienced heart failure, and 44.4% of patients had hemorrhage. Also, the mortality of RP‐ILD approaching 40% is consistent with the observed rates in other similar studies.^2^ However, the mortality of RP‐ILD with MAS was significantly higher nearly 88.9% (Table 5). These results suggest that MAS‐induced cytokine storms may lead to multiple organ failure in patients with RP‐ILD.

Our data suggest that the incidence of MAS in dermatomyositis may have been underestimated. The underestimation could be attributed to the rapid and severe mortality in patients with RP‐ILD, which might have led to the oversight of MAS as a comorbidity. Additionally, the relatively low prevalence of hemophagocytic cells in the diagnostic criteria may have contributed to this oversight. Our results indicate clinicians should be aware that when patients present with RP‐ILD, and along with abnormal laboratory findings, such as ferritin level >1685 ng/mL and hemoglobin <100 g/L, the presence of MAS should be considered in dermatomyositis. Investigating the relationship between MDA5 antibodies and MAS, as well as the involvement of various cytokines and macrophages in its pathogenesis, represents a potential direction for future research.

The main limitations of this study were the small sample size and its single‐center retrospective design. Another limitation of this study is the absence of myositis‐specific antibodies, especially MDA5 antibodies. Therefore, further prospective studies with larger sample sizes are required to validate our findings.

In conclusion, MAS was a common and fatal complication of adult dermatomyositis in our cohort, most cases of which were diagnosed during the initial stage of dermatomyositis. MAS in adult dermatomyositis occurred frequently in RP‐ILD patients. Patients with MAS more frequently developed multiple organ complications, resulting in a significant increase in the mortality rate.

## AUTHOR CONTRIBUTIONS

**Dingxian Zhu**: Writing—original draft; methodology; writing—review and editing; methodology. **Shunli Tang**: Data curation; writing—original draft. **Sheng Li, Changyi Yang, and Chuanyin Sun**: Validation; formal analysis. **Hong Fang and Jianjun Qiao**: Conceptualization; methodology; writing—review and editing.

## CONFLICT OF INTEREST STATEMENT

The authors declare no conflict of interest.

## ETHICS STATEMENT

Reviewed by institutional review boards of the First Affiliated Hospital, Zhejiang University School of Medicine (approval no: 2020‐ITT‐775).
